# Fumed-Si-Pr-Ald-Barb as a Fluorescent Chemosensor for the Hg^2+^ Detection and Cr_2_O_7_^2−^ Ions: A Combined Experimental and Computational Perspective

**DOI:** 10.3390/molecules29204825

**Published:** 2024-10-11

**Authors:** Ghodsi Mohammadi Ziarani, Mahtab Rezakhani, Mehran Feizi-Dehnayebi, Stoyanka Nikolova

**Affiliations:** 1 Department of Organic Chemistry, Faculty of Chemistry, Alzahra University, P.O. Box 19938-93973 Tehran, Iran; 2 Department of Organic Chemistry, Faculty of Chemistry, University of Plovdiv Paisii Hilendarski, 4000 Plovdiv, Bulgaria

**Keywords:** fumed-Si-Pr-Ald-Barb, fumed silica, chemosensor, fluorescent chemosensor, Hg^2+^, Cr_2_O_7_^2−^ ions, DFT

## Abstract

The surface of fumed silica nanoparticles was modified by pyridine carbaldehyde and barbituric acid to provide fumed-Si-Pr-Ald-Barb. The structure was identified and investigated through diverse techniques, such as FT-IR, EDX, Mapping, BET, XRD, SEM, and TGA. This nanocomposite was used to detect different cations and anions in a mixture of H_2_O:EtOH. The results showed that fumed-Si-Pr-Ald-Barb can selectively detect Hg^2+^ and Cr_2_O_7_^2−^ ions. The detection limits were calculated at about 5.4 × 10^−3^ M for Hg^2+^ and 3.3 × 10^−3^ M for Cr_2_O_7_^2−^ ions. A computational method (DFT) was applied to determine the active sites on the Pr-Ald-Barb for electrophilic and nucleophilic attacks. The HOMO-LUMO molecular orbital was calculated by B3LYP/6-311G(d,p)/LANL2DZ theoretical methods. The energy gap for the Pr-Ald-Barb and Pr-Ald-Barb+ion complexes was predicted by the E_HOMO_ and E_LUMO_ values. The DFT calculation confirms the suggested experimental mechanism for interacting the Pr-Ald-Barb with ions.

## 1. Introduction

Silicon dioxide (SiO_2)_ is mainly found in the earth’s crust. Silica exists in nature as a free element or combination with other oxides, usually found in quartz, clay, and sand [1]. Fused quartz, silica fume, and silica gel are compounds composed of silica for use in the food industry, drug delivery, microelectronics, and construction materials [2]. Nanosilica is also used in medicine as a poison-absorbing agent. In general, silicas, based on the origin of their production, are classified into natural silica, silica by-products, and synthetic silica. Natural silicas are quartz powder or diatom shells, while silica by-products include fused silica and fumed silica, which are by-products of factories and metallurgical industries and are mainly seen as micron-sized crystalline silicas. Sodium silicate, silicon tetrachloride, and alkoxy silanes are among the raw materials in the synthesis of silicas. The wet process is based on the reaction of silicates dissolved in the solvent, which produces silica gel. Due to the many features and benefits of using fumed silica, it has many applications in the industry and does not harm the environment. Fumed silica has many uses in the concrete and cement industry, in polymer applications, refractories, production of various types of silicates, as coating powders and insulating powders, auxiliary materials in the catalytic protection system, as a selective absorber of ions in the chemical industry, and as a catalyst in chemical reactions [3]. Silica has a very large, smooth, uneven surface that can make strong physical contact with functional groups. The surface properties of fumed silica are related to Si-O-Si units and silanol (OH-Si) groups, which are formed on the silica surface under the influence of air humidity at room temperature. Silanol group density strongly depends on the SiO_2_ production process [4].

In general, the surface of fumed silica nanoparticles can be modified by two methods: chemical and physical processes [5,6]. Modification is by physical interactions through the surfactants or macromolecules adsorbed on silica nanoparticles. A surfactant polar group or macromolecule is attracted to the silica surface by electrostatic interaction. This attraction can be controlled by different active surface materials [7]. Chemically modifying the nano-silica surface creates a stronger interaction between the modifiers and the silica nanoparticles. This includes modification either with modifiers or by grafting polymers, where the coupling of silane groups is one of the most widely used modifiers. The general structure of these groups is RSiX_3_, where X is the leaving group [8].

The organic group R can have different properties depending on the selected polymer. X groups react with hydroxyl groups on the surface of SiO_2_, and the R group interacts with arbitrary organic groups. Some of these compounds are 3-isocyanatopropylethoxysilane (ICPTES) with the formula OCN(CH_2_)_3_Si(OC_2_H_5_)_3_ and aminopropylmethyldiethoxysilane (APMDES) with the formula H_2_N(CH_2_)_3_(CH_3_)Si(OC_2_H_5_)_2_. In the following, articles on the applications of fumed silica in chemical reactions were studied [5]. In 2019, there was a report on the modification of fumed-Si-Pr-Cl with isonicotinic acid hydrazide (INAH) to yield fumed-Si-Pr-INAH, which was designed for the detection of mercury ions in fish samples [9].

Pollution is a thoughtful subject in the 21st century [10], which must be considered by researchers [11]. Due to their dangerous nature, oxide anions, such as Cr_2_O_7_^2−^, have received more research attention from scientists [12]. Cr_2_O_7_^2−^ is used in electroplating, paint, leather tanning, the glass industry, photography, and pesticides [13,14]. Cr_2_O_7_^2−^ anion is known as a heavy metal capable of causing ulceration and perforation of the nasal septum, kidney failure, and tumors [15,16]. Mercury ion (Hg^2+^) is one of the toxic pollutants whose continuous exposure, even at very low concentrations, can cause impairment of neurological functions such as movement and vision [6,7]. In addition, according to the World Health Organization (WHO), mercury can harm children’s nervous systems and brain development. It is not easy to recognize oxoanions such as Cr_2_O_7_^2−^ in the aqueous system because of their inherent characteristics. These types of anions have different charges and geometric shapes and can bind to water molecules. Therefore, new materials must be prepared for their selective detection. Optical sensor chemistry has the distinct advantages of simple operation, high sensitivity, non-degradation of the sample, and convenience of studies in living organisms. Therefore, in recent years, it has become an excellent option for the detection of metal ions [17,18]. The present fluorescent chemosensor has applications in environmental monitoring for detecting pollutants, biomedical diagnostics for disease detection and imaging, and food safety by identifying contaminants. It is also used in industrial quality control, water treatment, security for detecting hazardous substances, and pharmaceutical research, providing sensitive and specific real-time analysis across these fields.

Continuing our previous research, we examined and characterized the optical properties of chemosensors toward different anions and cations [9]. In this attempt, fumed-Si-Pr-Ald-Barb was synthesized, and its optical activity was studied.

## 2. Results and Discussion

**Scheme 1:**
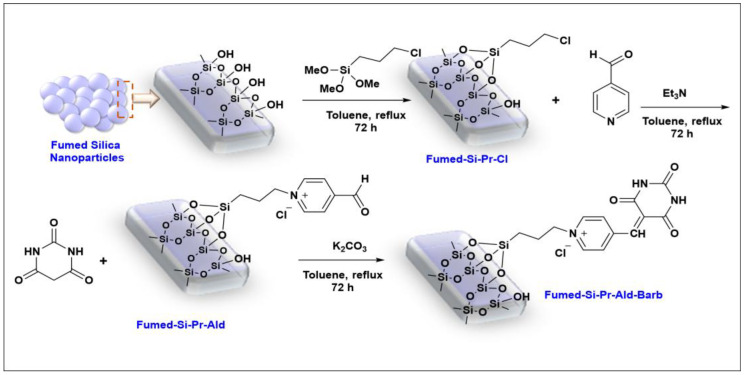
Synthesis of fumed-Si-Pr-Ald-Barb.

At first, the fumed-Si-Pr-Cl surface was modified with pyridinecarbaldehyde to obtain fumed-Si-Pr-Ald, which was in turn functionalized with barbituric acid using potassium carbonate in toluene to produce fumed-Si-Pr-Ald-Barb (Scheme 1).

**Scheme 2:**
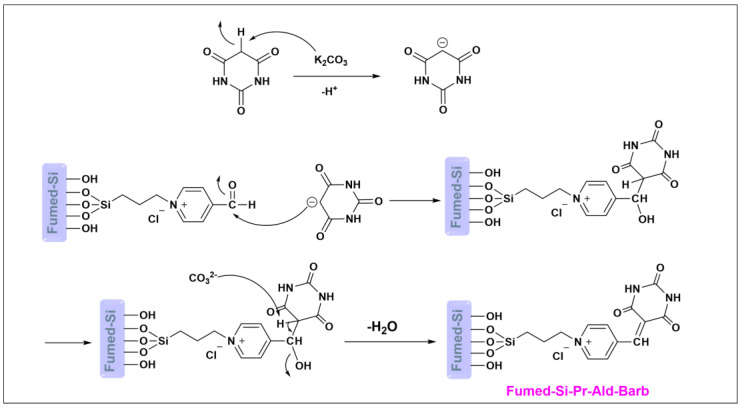
The mechanism of fumed-Si-Pr-Ald-Barb.

The synthesis mechanism of fumed-Si-Pr-Ald-Barb was shown in Scheme 2. Initially, the K_2_CO_3_ base separates the acidic hydrogen of barbituric acid, and the resulting anion is attached to the carbonyl group of fumed-Si-Pr-Ald to give fumed-Si-Pr-Ald-Barb by dehydrating.

### 2.1. Characterization

#### 2.1.1. FT-IR Spectroscopy

**Figure 1:**
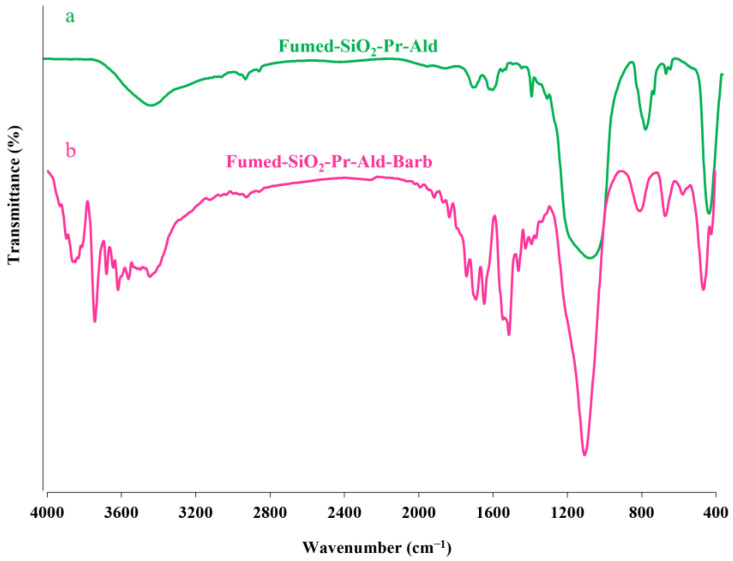
(a) Fumed-Si-Pr-Ald; (b) Fumed-Si-Pr-Ald-Barb.

The obtained results of the FT-IR spectrum related to fumed-Si-Pr-Ald-Barb composition are shown in Figure 1. The absorption band in the areas of 3400 and 3700 cm^−1^ is related to the stretching vibrations of silanol-OH groups on the surface of fumed silica (presence of hydrogen bonding).

The absorption band in the areas of 2854 cm^−1^ and 2925 cm^−1^ is related to the stretching vibrations of methylene groups (-CH_2_) in the propyl chain, which indicates the functionalization by (3-chloropropyl)trimethoxysilane groups on the surface of fumed-S (presence of hydrogen bonding). The absorption band of the stretching vibrations of the C-N bond in the barbituric acid ring corresponding to spectrum b in the region of about 1200 cm^−1^ to 1300 cm^−1^ has overlapped with the stretching vibrations of the Si-O-Si and Si-OH bonds. The absorption band appearing in the region of 1720 cm^−1^ is related to the carbonyl aldehyde group of the pyridine carbaldehyde molecule. The absorption band in the region of 1650 cm^−1^ and 1690 cm^−1^, which are two branches in peak b, is the interference of the N-H bending band with the carbonyl C=O amide group of barbituric acid. The absorption band in the area of 1500 cm^−1^ and 3450 cm^−1^ is related to the bending and stretching vibrations of the N-H bond of spectrum b. The absorption band appearing in the region of 1600 cm^−1^ and 1620 cm^−1^ is related to C=N and C=C, respectively. The absorption band in the area of 800 and 1100 cm^−1^ in both spectra is related to the symmetric stretching vibrations of Si-O-Si and Si-OH bonds.

#### 2.1.2. EDX Studies

By using the X-ray diffraction spectroscopic analysis technique, it is possible to find out the composition of the elements in fumed silica and the weight percentage of each element. According to Figure S1, all the elements, including Si, C, O, and N, are present in the nanoparticle structure with percentages of 42.8, 32.2, 0.24, and 1.1, respectively.

#### 2.1.3. Mapping Image

**Figure 2:**
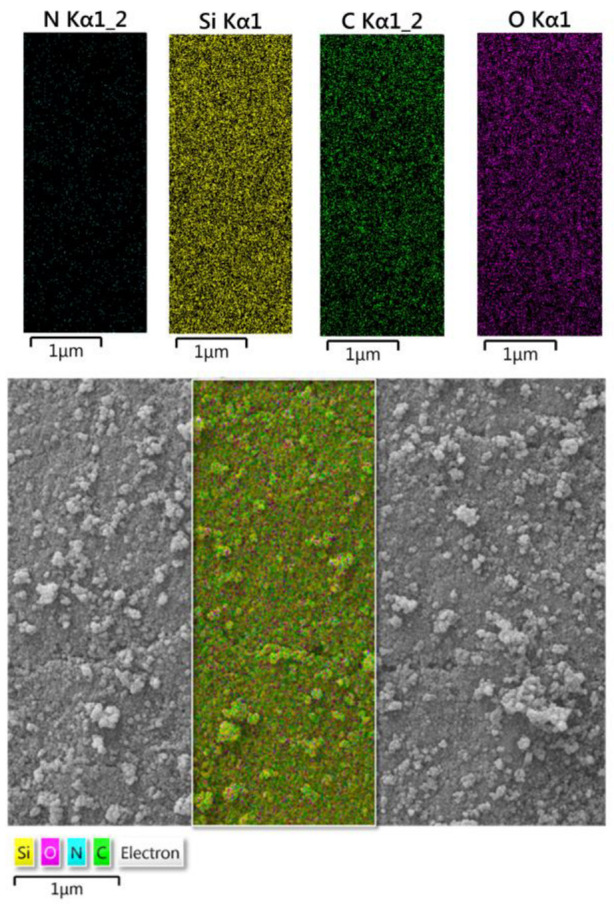
Mapping of fumed-Si-Pr-Ald-Barb.

Figure 2 shows the mapping images of the fumed-Si-Pr-Ald-Barb composition. These images show the distribution of elements in the EDX investigated area, and according to these images, the uniform distribution of silicon, carbon, oxygen, and nitrogen elements on the surface of the fumed silica is noticeable.

#### 2.1.4. N_2_ Adsorption-Desorption Analysis

**Figure 3:**
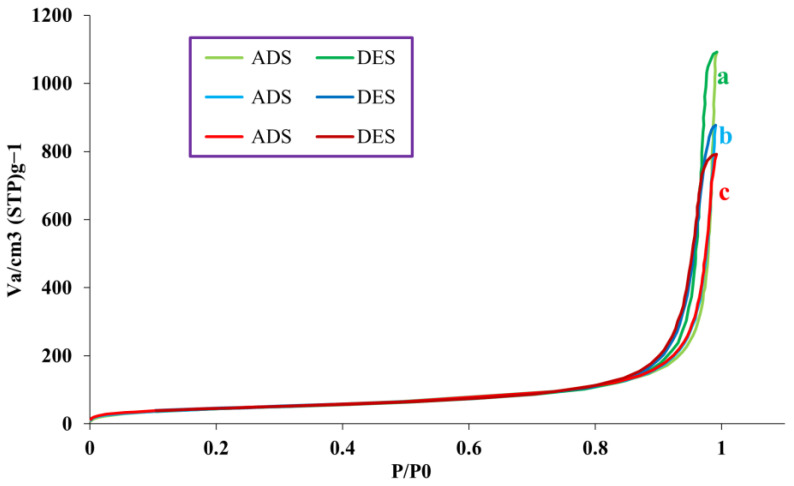
N_2_ adsorption-desorption for (a) Fumed-Si-Pr-Cl; (b) Fumed-Si-Pr-Ald; (c) Fumed-Si-Pr-Ald-Barb.

Nitrogen adsorption-desorption diagrams to measure the surface and size of nanoparticles of fumed-Si-Pr-Cl, fumed-Si-Pr-Ald, and fumed-Si-Pr-Ald-Barb compounds are shown in Figure 3. These samples have type II isotherms, which are related to non-porous compounds. This behavior indicates that the material exhibits multilayer adsorption on a smooth surface without the complexities associated with pore filling or hysteresis. Decreasing the values of structural parameters indicates the settling of organic species on the surface.

Three parameters were reduced: the specific surface area (S_BET_), total pore volume (V_Total_), and pore size [19] for the fumed-Si-Pr-Cl, fumed-Si-Pr-Ald, and fumed-Si-Pr-Ald-Barb, thus the effective conjunction on the fumed-Si-Pr-Cl (Table 1).

#### 2.1.5. SEM Studies

**Figure 4:**
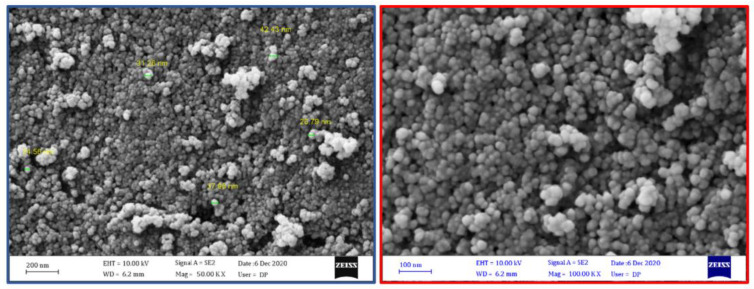
SEM of fumed-Si-Pr-Ald-Barb.

SEM image of the fumed-Si-Pr-Ald-Barb composition showed masses with a spherical structure due to the morphology of fumed silica (Figure 4). The average size of the spherical nanoparticles is 36 nm. From the SEM image, we can conclude that the structure and morphology of fumed silicas did not change during the surface reaction and functionalization.

#### 2.1.6. TGA Studies

**Figure 5:**
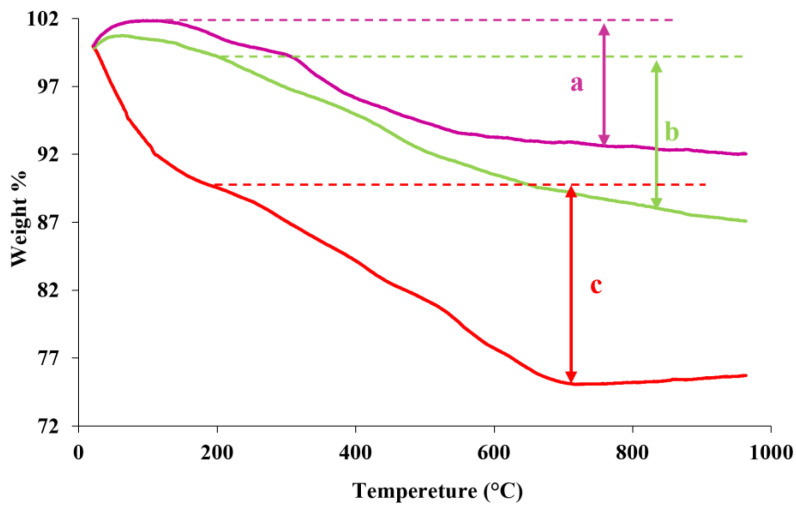
TGA analysis of (a) Fumed-Si-Pr-Cl; (b) Fumed-Si-Pr-Ald; (c) Fumed-Si-Pr-Ald-Barb.

This analysis evaluated the number of organic groups placed on the surface. The curves related to the thermogravimetric analysis of fumed-Si-Pr-Cl, fumed-Si-Pr-Ald, and fumed-Si-Pr-Ald-Barb samples are shown in Figure 5. The weight loss of samples up to 200 °C is related to the removal of H_2_O absorbed on the samples and the range of 200–800 °C to the destruction of organic species. The weight loss above 800 °C is related to the hydroxylation of the Si-OH on the surface of the fumed silica. Therefore, the loss weight percentage related to the devastation, the organic species of fumed-Si-Pr-Cl, fumed-Si-Pr-Ald, and fumed-Si-Pr-Ald-Barb products is 10%, 11%, and 15%, respectively.

### 2.2. Fluorescent Studies

#### 2.2.1. Fluorescence Response Test for Cations

**Figure 6:**
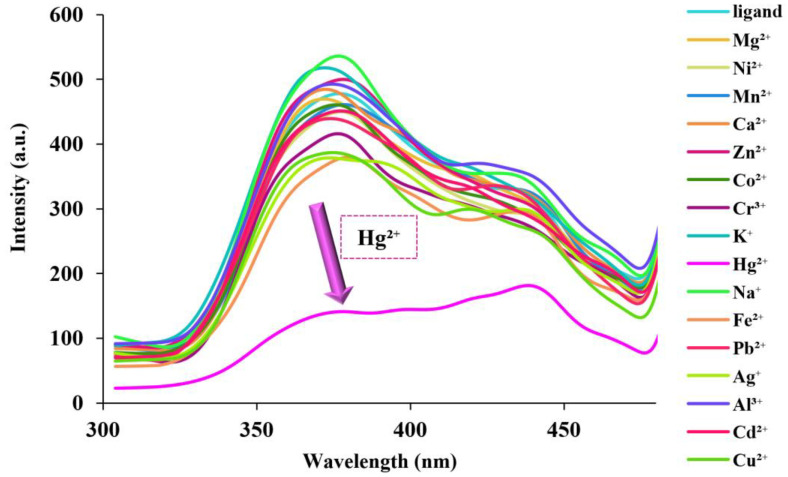
Fluorescence spectra of the aqueous suspended fumed-Si-Pr-Ald-Barb (2.5 mL, 0.02 g in 100 mL (H_2_O:EtOH/2:3)) by different cations (λₑ_m_ = 300 nm, λₑ_x_ = 375 nm).

The fluorescence response of fumed-Si-Pr-Ald-Barb by preparation of an aqueous solution with a concentration of 0.02 g in 100 mL (H_2_O:EtOH) at 300 nm is studied by adding diverse metal ions. The fumed-Si-Pr-Ald-Barb was provided by dissolving 0.02 g of fumed-Si-Pr-Ald-Barb (H_2_O:EtOH). The cation metal ions were gained by dissolving their nitrate salts in H_2_O (10^−2^ M). In this experiment, 2.5 mL suspension of fumed-Si-Pr-Ald-Barb was brought into a vial by adding 200 µL of metal ions solution, such as Ni^2+^, Mg^2+^, Zn^2+^, Ag^+^, Cd^2+^, Cr^3+^, Co^2+^, Cu^2+^, Al^3+^, Ca^2+^, Mn^2+^, Pb^2+^, Fe^2+^, Na^+^, K^+^, and Hg^2+^ for measurement. The results showed that the intensity of the fluorescence spectra of fumed-Si-Pr-Ald-Barb increased with the addition of the Hg^2+^ ion (Figure 6).

#### 2.2.2. Possible Mechanism of Bond Formation between Fumed-Si-Pr-Ald-Barb Compound and Hg^2+^ Ion

The possible mechanism of fumed-Si-Pr-Ald-Barb combination with mercury II ion is shown in Scheme S1. Mercury ion can interact with the pair of electrons of nitrogen and oxygen of barbituric acid. Mercury quenching is greater with some cations because they influence the electronic environment or binding efficiency of mercury to the chemosensor. Factors like charge density, ionic radius, and coordination chemistry can enhance mercury-cation interactions, increasing quenching efficiency.

#### 2.2.3. Competition Test

The selectivity of the chemosensor for Hg^2^⁺ in competition with other cations is shown in Figure S2. Fumed-Si-Pr-Ald-Barb (2.5 mL, 0.02 g in 100 mL (H_2_O:EtOH)) and other cations (200 µL, 1 × 10^−2^ M) were mixed with Hg^2^⁺ (200 µL, 1 × 10^−2^ M) to conduct a competition test. The results indicated that fumed-Si-Pr-Ald-Barb is a chemosensor for Hg^2^⁺ ions.

#### 2.2.4. Titration Test

**Figure 7:**
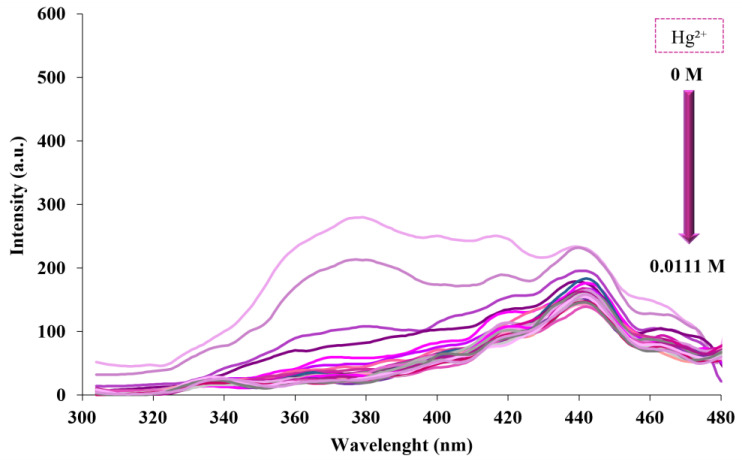
Fluorescence response of fumed-Si-Pr-Ald-Barb after adding different concentrations of Hg^2+^ (10, 20, …, 300 µL) (λₑ_m_ = 300 nm, λₑ_x_ = 375 nm).

Titration spectra were documented by adding different amounts of Hg^2+^ (10^−2^ M) to the aqueous suspension (2.5 mL) of fumed-Si-Pr-Ald-Barb. The relationship between fluorescence intensity and different concentrations of Hg^2+^ is demonstrated in Figure 7.

A fluorescence titration test was performed to calculate the detection limit. The plot of the fluorescence intensity of fumed-Si-Pr-Ald-Barb against different concentrations of Hg^2+^ proves the linear relation between them: y = −2779.3x + 97.929.

Regression coefficient: R^2^ = 0.961

Then, according to the DL = 3Sd/m equation, DL: 5.4 × 10^−3^ M (Figure S3).

#### 2.2.5. Fluorescence Response Test for Anions

**Figure 8:**
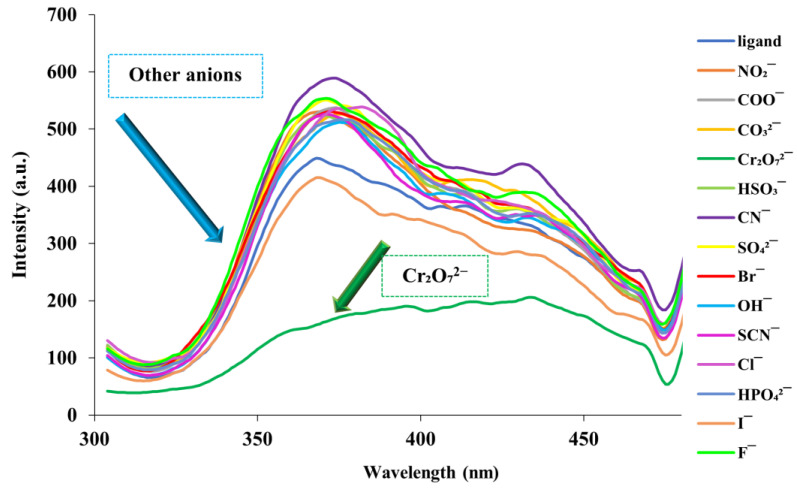
Fluorescence spectra of the aqueous suspended fumed-Si-Pr-Ald-Barb (2.5 mL, 0.02 g in 100 mL (H_2_O:EtOH/2:3)) by different anions (λₑ_m_ = 300 nm, λₑ_x_ = 380 nm).

Fumed-Si-Pr-Ald-Barb combination against various anions (including CO_3_^2−^, Cl^−^, CN^−^, SCN^−^, Br^−^, F^−^, OH^−^, I^−^, Cr_2_O_7_^2−^, HSO_3_^−^, SO_4_^2−^, CH_3_COO^−^, HPO_4_^2−^, NO_2_^−^, and NO_3_^−^) were investigated. In this way, 0.02 g of fumed-Si-Pr-Ald-Barb was dispersed in 100 mL of water/ethanol (2:3) solvent. Then, 2.5 mL of the dispersed solution was added to 200 microliters of the mentioned anions with a concentration of 0.01 M, and the fluorescence emission of each was checked. The results obtained from the corresponding diagram (Figure 8) indicate that the fluorescence emission of the fumed-Si-Pr-Ald-Barb decreases in the presence of Cr₂O₇^2−^ ion.

#### 2.2.6. Competitive Test of Cr₂O₇^2−^ Ion with Other Anions

A competitive test of the fumed-Si-Pr-Ald-Barb was performed to identify Cr₂O₇^2−^ ion. To perform this test, we added 200 microliters of Cr₂O₇^2−^ ion to other anions, and their fluorescence emission spectrum was checked. The obtained data show that the fumed-Si-Pr-Ald-Barb compound has sense properties in the presence of Cr₂O₇^2−^ ion (200 µL) (Figure S4).

**Figure 9:**
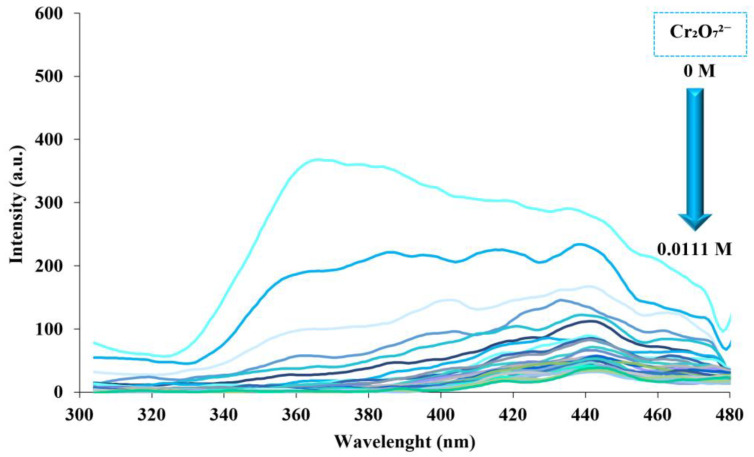
Fluorescence response of fumed-Si-Pr-Ald-Barb after adding different concentrations of Cr₂O₇^2−^ (10, 20, …, 300 µL) (λₑ_m_ = 300 nm, λₑ_x_ = 380 nm).

We investigated the fluorescence spectrum of Fumed-Si-Pr-Ald-Barb composition against different Cr₂O₇^2−^ ions. Titration was performed by adding 100 microliters of Cr₂O₇^2−^ ion to the ligand each time to assess the effect of different concentrations of Cr₂O₇^2−^ ion. According to the examination of fluorescence spectrum data, with the increase of Cr₂O₇^2−^ ion concentration, the emission intensity of fumed-Si-Pr-Ald-Barb composition decreases (Figure 9).

The linear relationship between Cr₂O₇^2−^ ion concentration and emission intensity of the fumed-Si-Pr-Ald-Barb compound is shown in Figure S5. The regression coefficient was 0.97, and the detection limit of this compound was calculated with the formula DL = 3Sd/m, and its value was determined as 3.3 × 10^−3^ M.

#### 2.2.7. Possible Mechanism of Bond Formation between Fumed-Si-Pr-Ald-Barb Compound and Cr₂O₇^2−^ Ion

The possible mechanism of fumed-Si-Pr-Ald-Barb combination with dichromate ion is shown in Scheme S2. Since the pyridinium carbaldehyde ring has a positive charge, it can absorb the dichromate anion and replace the chlorine ion. When the wavelength excites the Si-Pr-Ald-Barb material, the emission from Si-Pr-Ald-Barb coincides with the absorption peak of Cr₂O₇^2^⁻ ion. As a result, energy transfer occurs from the material to the dichromate ions, leading to quenching. This overlap between the emission wavelength of Si-Pr-Ald-Barb and the absorption peak of Cr₂O₇^2^⁻ is the key reason for the observed quenching behavior [20].

#### 2.2.8. Comparison

The synthesized sensor was compared with other reported chemosensors, which illustrated the selectivity and sensitivity of our sensors compared to others (Table 2).

### 2.3. Computational Details

#### 2.3.1. DFT Base Mechanism

Because the surface lacks sensor properties, we concentrated our calculations solely on the ligand (organic) component. The DFT theoretical approach for the ground-state structure was carried out using 6-311G(d,p) (for H, N, C, Cl, and O atoms)/LANL2DZ basis set (for Cr and Hg ions). Using GaussView 6.0 software, the initial geometries were designed and then input into Gaussian 09W. The Gaussian output was analyzed to identify potential interaction sites and for additional quantum assessments. All theoretical analyses for the ground-state geometry of Pr-Ald-Barb, Pr-Ald-Barb+Hg^2+^, and Pr-Ald-Barb+Cr_2_O_7_^2−^ were obtained through the same level of calculation. All generated geometries display positive frequencies, confirming that all structures are at real minima [23]. By employing the DFT approach, we can pinpoint the precise binding sites on Pr-Ald-Barb for the Hg^2+^ and Cr_2_O_7_^2−^ ions. To uncover this truth, we analyzed the geometry optimization, MEP map, molecular orbital theory, and quantum reactivity parameters for Pr-Ald-Barb, Pr-Ald-Barb+Hg^2+^, and Pr-Ald-Barb+Cr_2_O_7_^2−^.

**Figure 10:**
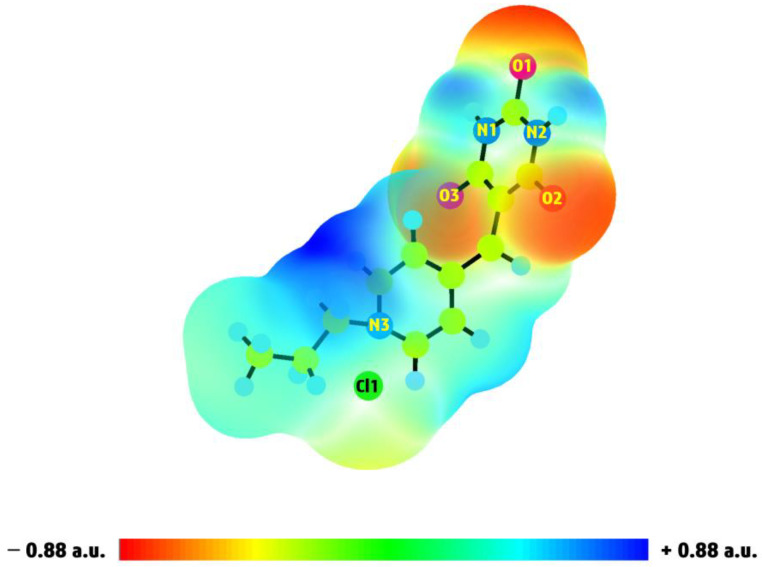
The MEP surface of Pr-Ald-Barb generated from DFT calculation.

The molecular electrostatic potential (MEP) is a crucial tool for elucidating the arrangement of structural components on the molecular surface. Utilizing MEP, the active sites on Pr-Ald-Barb for electrophilic and nucleophilic reactions can be determined [24]. These active regions are used to pinpoint areas involved in biological activity, hydrogen bonding, catalytic functions, and interactions with ions. The MEP map uses color to show the potential for electrostatic charges around the chemical geometry of a molecule. This map illustrates the electrostatic potential of the molecule, with positive values depicted in blue and negative values shown in red. Generally, blue surfaces are suitable for nucleophilic attacks, while red surfaces are appropriate for electrophilic attacks [25]. The red area is electron-rich and tends to engage with positively charged ions. The MEP surface of the Pr-Ald-Barb molecule was also estimated using its optimized geometry with the 6-311G(d,p) basis set, and the result is demonstrated in Figure 10. In the MEP of Pr-Ald-Barb, the blue regions around the hydrogen atoms bonded to N1, N2, and certain carbon atoms indicate nucleophilic sites that are likely to form coordinated bonds with receptor macromolecules, potentially enhancing bioactivity. Red zones (O1, O2, and O3 atoms) signify a higher negative charge, increased electron density, and a greater tendency to attract positive charges (protons). As a result, the MEP surface indicates that the regions surrounding the O1, O2, and O3 atoms are optimal for interacting with the Hg^2+^ ion. The color scheme of the MEP map for Pr-Ald-Barb varies from −0.88 a.u. (red surface) to +0.88 a.u. (blue surface).

**Figure 11:**
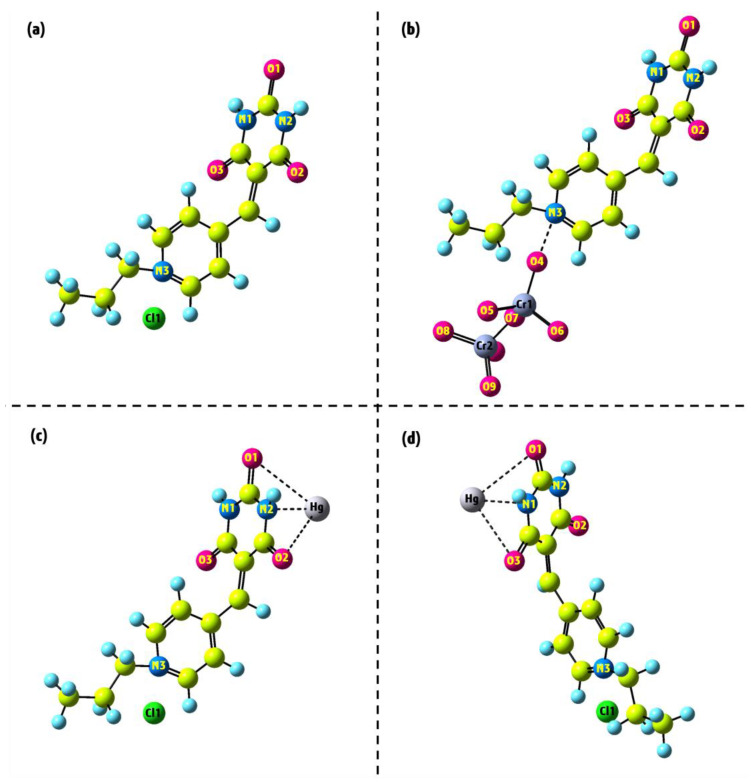
The optimized geometries of (**a**) Pr-Ald-Barb; (**b**) Pr-Ald-Barb+Cr_2_O_7_^2−^; (**c**,**d**) Pr-Ald-Barb+Hg^2+^ under the DFT approach.

Given that the MEP surface suggests the regions around the O1, O2, and O3 atoms are favorable for binding with the Hg^2+^ ion, we place this ion in various positions within these areas. Various geometries for the Pr-Ald-Barb+Hg^2+^ complex have been optimized, and the results indicate that the optimal position for the Hg^2+^ ion interaction is situated between the O1, N2, and O2 atoms with an electronic energy of −1396.28 Hartree, as depicted in Figure 11c. Another position, located near the O1, N1, and O3 atoms, has an electronic energy of −1395.99 Hartree, as shown in Figure 11d. This result confirms that the Hg^2+^ ion binds more stably with the O1, N2, and O2 atoms.

To determine the optimal position for the interaction between Cr_2_O_7_^2−^ and Pr-Ald-Barb, we first substituted the Cr_2_O_7_^2−^ ion with a Cl atom in the structure and then conducted the geometry optimization. The result demonstrated that this ion in the mentioned position has better electronic energy (−1592.61 Hartree) compared to other positions, thereby supporting the proposed experimental interaction mechanism (Figure 11b). Thus, the MEP diagram and geometry optimization confirm the proposed mechanism for the interaction between Pr-Ald-Barb and Hg^2^⁺ as well as Cr₂O₇^2−^, as represented in Schemes S1 and S2.

#### 2.3.2. Mulliken Charge Distribution

The Mulliken atomic charge reveals the net positive or negative charge of each atom in a molecule, making it one of the most commonly utilized methods for population analysis. The distribution of atomic charges influences several molecular properties, including polarizability, dipole moments, and electronic energy [26]. The atomic net charges obtained through the Mulliken method for the selected atoms of Pr-Ald-Barb are calculated and presented in Table S1. The net atomic charges calculated for the O1 (−0.48), O2 (−0.47), O3 (−0.46), N1 (−0.336), and N2 (−0.340) atoms are negative, indicating that these regions are suitable for electrophilic reactions. As a result, these atoms are likely to interact with positively charged species, such as Hg^2+^. Moreover, the net atomic charges on the N3 atom and the hydrogen atoms bonded to N1 and N2 (H_-N1_ and H_-N2_) are calculated to be +0.41, +0.28, and +0.28, respectively. Consequently, these atoms are expected to interact with negatively charged species, such as Cr_2_O_7_^2−^.

#### 2.3.3. Fukui Function and Dual Descriptor Analysis

The Fukui function is a local density functional tool used to identify reactive regions within compounds. It highlights specific areas where the electron density of chemical species shifts as the electron count changes [27]. The Fukui function provides values for nucleophilic (f^+^) and electrophilic (f^−^) attacks, which are calculated using the following Formulas (1) and (2):

(1) $$f^{+}=q\left(N+1\right)-q\left(N\right)\;$$

(2) $$f^{-}=q\left(N\right)-q\left(N-1\right)$$

In this context, q(N + 1), q(N), and q(N − 1) denote the Mulliken charge of an atom with N + 1, N, and N − 1 electrons, respectively. The electrons in the anionic, neutral, and cationic states are represented by the chemical species corresponding to N + 1, N, and N − 1. Furthermore, the dual descriptor (Δ*f*) integrates data from the Fukui functions to reflect both nucleophilic and electrophilic characteristics at a particular site according to the following Equation (3):

(3) $$\Delta f=f^{+}-f^{-}$$

To gain deeper insights into the structural characteristics of Pr-Ald-Barb following the addition of Hg^2^⁺ and Cr₂O₇^2^⁻, the proper sites for electrophilic and nucleophilic reactions were identified through the calculation of the condensed dual descriptor, a method recognized as highly reliable for predicting site reactivity [28]. The Fukui indices and dual descriptors for the selected atoms were assessed using the B3LYP/6-311G (d,p) computational level, and the findings are presented in Table S2. For simplicity, the analysis focused solely on the Fukui functions and dual descriptors for the O1, O2, O3, N1, N2, N3, H_-N1_, and H_-N2_ atoms. We can evaluate the likelihood of these atoms serving as reactive sites by considering the value of Δ*f*. Generally, a positive value of Δ*f* suggests that the site is conducive to nucleophilic attack, whereas a negative value indicates that it is more favorable for electrophilic attack [29]. Data indicates that the distribution of Δ*f* around the N2 and O2 atoms is more negative than N1 and O3, suggesting that the region around the O1, N2, and O2 are conducive to electrophilic attack and can effectively interact with Hg^2^⁺. On the other hand, the distribution of Δ*f* around the N3 atom is more positively charged compared to other regions, indicating that this area is favorable for nucleophilic attack and can effectively engage with Cr₂O₇^2^⁻. The results obtained from the Fukui function and dual descriptor are consistent with the analysis of the MEP surface and Mulliken atomic charges.

#### 2.3.4. Molecular Orbital Theory

**Figure 12:**
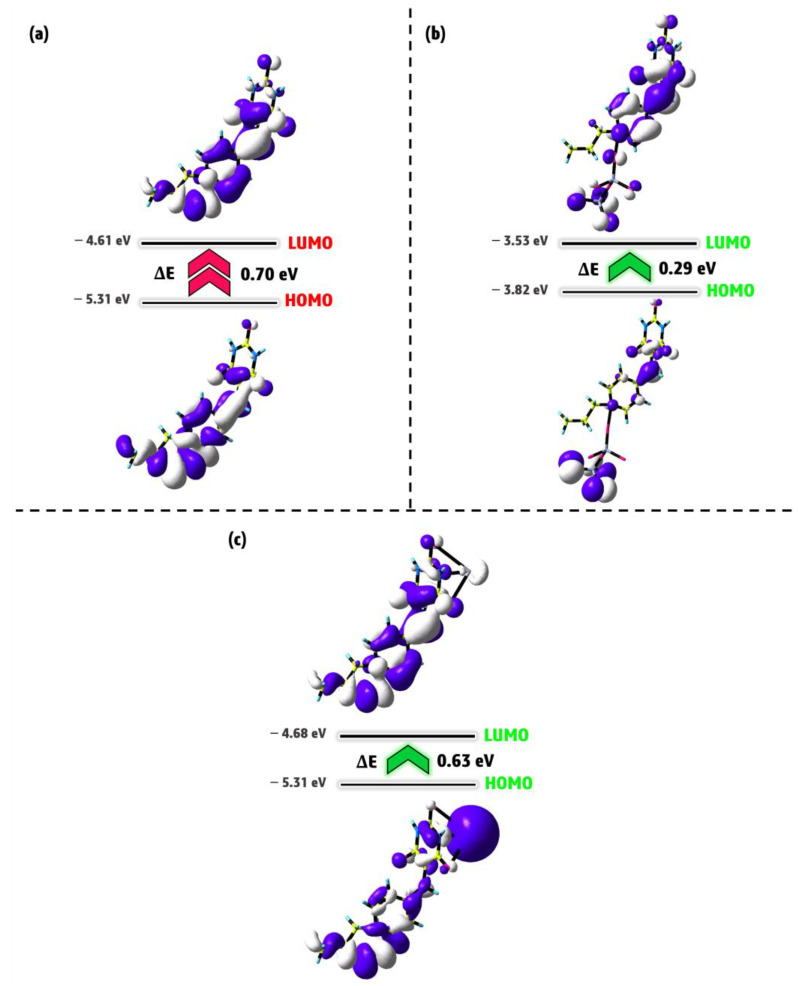
The FMOs distribution of (**a**) Pr-Ald-Barb, (**b**) Pr-Ald-Barb+Cr_2_O_7_^2−^, and (**c**) Pr-Ald-Barb+Hg^2+^.

To gain a clearer understanding of electron delocalization and the sensing behavior of Pr-Ald-Barb toward Hg^2+^ and Cr_2_O_7_^2−^, we systematically performed molecular orbital theory calculations using DFT both before and after complexation with these ions. The frontier molecular orbitals (FMOs) consist of the HOMO and LUMO. The HOMO relates to the ionization potential and serves as an electron donor. In contrast, the LUMO is connected to electron affinity and acts as an electron acceptor [30]. An analysis of the contour plots for significant FMO shows that, for Pr-Ald-Barb, the charge is distributed across the entire ligand at both the HOMO and LUMO levels. In the Pr-Ald-Barb+Cr_2_O_7_^2−^ and Pr-Ald-Barb+Hg^2+^ complexes, the electronic cloud of the HOMO is predominantly associated with Hg^2+^, Cr_2_O_7_^2−^, and to a lesser extent with the ligand, while the electronic cloud of the LUMO is primarily concentrated on the Pr-Ald-Barb portion (Figure 12). The energy gaps between HOMO-LUMO (∆E) are found to be 0.70, 0.29, and 0.63 eV for Pr-Ald-Barb, Ald-Barb+Cr_2_O_7_^2−^, and Pr-Ald-Barb+Hg^2+^, respectively. The decrease in ∆E value upon the formation of the complex between Pr-Ald-Barb and Hg^2+^ or Cr_2_O_7_^2−^ indicates an increase in the chemical reactivity of Pr-Ald-Barb when interacting with these ions [31]. The HOMO and LUMO orbital levels of the Pr-Ald-Barb+Cr_2_O_7_^2−^ and Pr-Ald-Barb+Hg^2+^ complexes are very close to each other, indicating that the presence of Hg^2+^ and Cr_2_O_7_^2−^ ions enhances the reactivity of the compound compared to free Pr-Ald-Barb. This is because the close proximity of the HOMO and LUMO levels facilitates easier electron transitions to the higher energy state [32].

The quantum properties of Pr-Ald-Barb, Pr-Ald-Barb+Hg^2+^, and Pr-Ald-Barb+Cr_2_O_7_^2−^, including absolute softness (σ = 1/η), chemical potential (P_i_ = −χ), absolute electronegativity (χ = −(E_HOMO_ + E_LUMO_)/2), absolute hardness (η = (E_LUMO_ − E_HOMO_)/2), and global electrophilicity (ω = P_i_^2^/2η) were examined using E_HOMO_ and E_LUMO_, as summarized in Table S3 [33]. These parameters act as measures of a compound’s stability and reactivity. A smaller bandgap value signifies that a compound is softer and more reactive. Among these, the Pr-Ald-Barb+Hg^2+^ and Pr-Ald-Barb+Cr_2_O_7_^2−^ complexes are considered softer and more reactive compared to the free Pr-Ald-Barb. The negative value of the chemical potential indicates that these complexes are in a stable state.

#### 2.3.5. Reduced Density Gradient (RDG) Calculation

**Figure 13:**
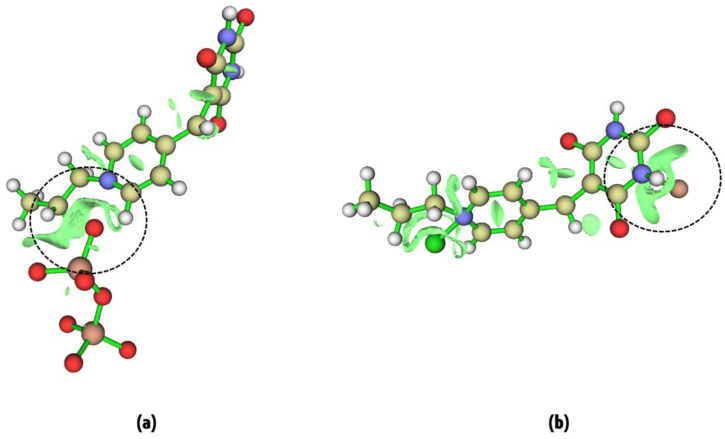
RDG surface of (**a**) Pr-Ald-Barb+Cr_2_O_7_^2−^ and (**b**) Pr-Ald-Barb+Hg^2+^.

In order to gain deeper insights into the intermolecular interactions responsible for the chemosensor activity of the ligand with Hg^2^⁺ and Cr₂O₇^2^⁻ ions, we performed an RDG analysis. The RDG method is a well-established approach for visualizing non-covalent interactions (NCIs), such as van der Waals forces, hydrogen bonding, and electrostatic interactions, which are crucial in determining the binding affinity and selectivity of chemosensors. RDG is particularly useful because it highlights regions where these weak interactions occur by mapping them as surfaces with different colors, depending on their nature and strength. The RDG analysis for Pr-Ald-Barb+Cr_2_O_7_^2−^ and Pr-Ald-Barb+Hg^2+^ is represented in Figure 13. In Pr-Ald-Barb+Cr_2_O_7_^2−^, a significant green surface appears between Cr_2_O_7_^2−^ and Pr-Ald-Barb, indicating the presence of strong interactions in this region. In this case, the O4 atom of Cr_2_O_7_^2−^ has interacted with the N3 atom of Pr-Ald-Barb. In the Pr-Ald-Barb+Hg^2+^, a noticeable green surface emerges between Hg^2+^ and Pr-Ald-Barb, signifying strong interactions within this area. In this case, the Hg^2+^ ion interacts with the O1, N2, and O2 atoms of Pr-Ald-Barb. The green color corresponds to weakly attractive van der Waals interactions, which are key contributors to the binding of metal ions in many chemosensory systems. This large green surface suggests that Pr-Ald-Barb forms a stable complex with Cr_2_O_7_^2−^ and Hg^2+^, supported by these non-covalent forces. This broad surface implies a high degree of interaction between the metal ions and Pr-Ald-Barb, enhancing the stability and specificity of the chemosensor. These RDG results align with other computational results, further confirming the potential of Pr-Ald-Barb as an effective chemosensor for Cr_2_O_7_^2−^ and Hg^2+^ ion detection.

## 3. Materials and Methods

### 3.1. Surface Modification of Silica Fume with (3-Chloropropyl)trimethoxysilane

It was published previously [34].

### 3.2. Modification of Fumed-Si-Pr-Cl Surface with Pyridinecarbaldehyde

To dried fumed-Si-Pr-Cl (1 g) in dried toluene (50 mL), triethylamine (10 mmol, 1.3 mL) and pyridinecarbaldehyde (10 mL) were added and refluxed for 72 h to give fumed-Si-Pr-Ald-Barb, which was soxhelated with CH_3_OH.

### 3.3. Synthesis of Fumed-Si-Pr-Ald-Barb

Fumed-Si-Pr-Ald (0.8 g) was dispersed in dry toluene (30 mL), then potassium carbonate (0.02 g) and barbituric acid (5 mmol) were added and refluxed for one week. The resulting material was washed with H_2_O, EtOH, and acetone at room temperature. It was soxhelated with ethanol and dried at room temperature to gain fumed-Si-Pr-Ald-Barb, which was characterized.

### 3.4. Computational Details

A computational perspective was performed using GaussView 6.0/Gaussian 09W software [35] in order to verify the proposed mechanism of interaction by experimental methods. We used the DFT method along with the B3LYP functional level for geometry optimization. For this calculation, the LANL2DZ basis set was applied to the heavy atoms (Cr and Hg), whereas the 6-311G(d,p) basis set was used for the remaining atoms (H, N, C, Cl, and O). The potential interaction site with the ion was identified on the compound by investigating the molecular electrostatic potential (MEP) map. Chemical reactivity and stability were studied using molecular orbital theory. Additionally, the quantum reactivity parameters were determined based on the energy values of the HOMO and LUMO. The Mulliken charge distribution, Fukui function, and dual descriptor analysis were performed in order to confirm the binding sites of the ions on Pr-Ald-Barb. On the other hand, RDG calculations were conducted using Multiwfn software.

## 4. Conclusions

In this work, a fluorescent chemical sensor based on fumed silica was synthesized using a multicomponent reaction on the surface of nanoparticles. This sensor has an organic part of pyridinecarbaldehyde and barbituric acid. By examining the fluorescence effect of the fumed-Si-Pr-Ald-Barb compound against various cations and anions, the fluorescence intensity of this compound against Hg(II) and dichromate ions decreased significantly. Considering the importance of mercury ions and dichromate, this compound can be a very good indicator of these ions. All theoretical analyses for the ground-state geometry of Pr-Ald-Barb, Pr-Ald-Barb+Hg^2+^, and Pr-Ald-Barb+Cr_2_O_7_^2−^ were obtained through B3LYP/6-311G(d,p)/LANL2DZ level. The MEP diagram, geometry optimization, Mulliken charge distribution, Fukui function, and dual descriptor analysis along with RDG surface confirm the proposed mechanism for the interaction between Pr-Ald-Barb and Hg^2^⁺, as well as Cr₂O₇^2−^. The HOMO and LUMO orbital levels of the Pr-Ald-Barb+Cr_2_O_7_^2−^ and Pr-Ald-Barb+Hg^2+^ complexes indicated that the presence of Hg^2+^ and Cr_2_O_7_^2−^ ions enhances the reactivity of the complex compared to free Pr-Ald-Barb.

## Figures and Tables

**Table 1:** Textural properties of fumed-Si-Pr-Cl, fumed-Si-Pr-Ald, and fumed-Si-Pr-Ald-Barb.

Sample	S_BET_ (m^2^ g^−1^)	V_Total_ (cm^3^ g^−1^)	d_P_ [18]
Fumed-Si-Pr-Cl	155	0.370	36
Fumed-Si-Pr-Ald	153	0.421	37
Fumed-Si-Pr-Ald-Barb	149	0.429	38

**Table 2:** Comparison studies among different ligands towards Hg^2+^.

No	Chemosensor	DL	Media	Ref.
1	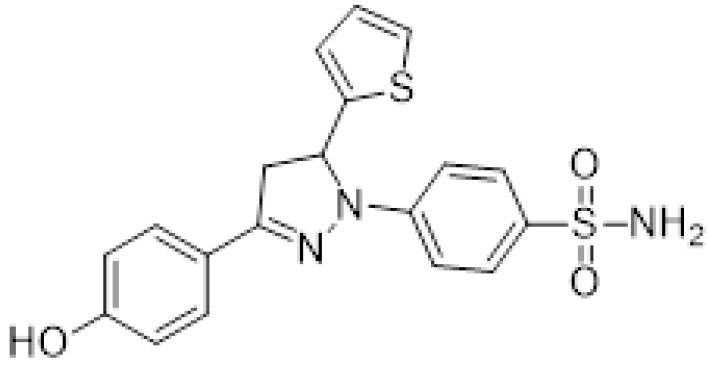	-------	DMF	[21]
2	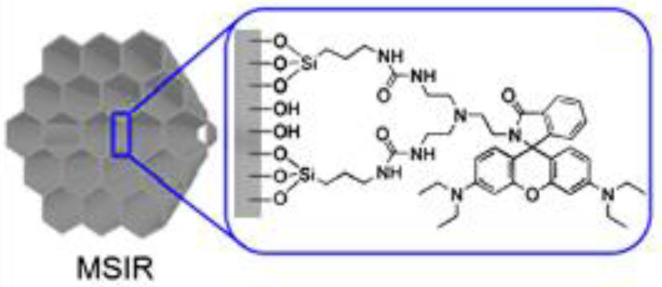	------------	CH_3_CN	[22]
3	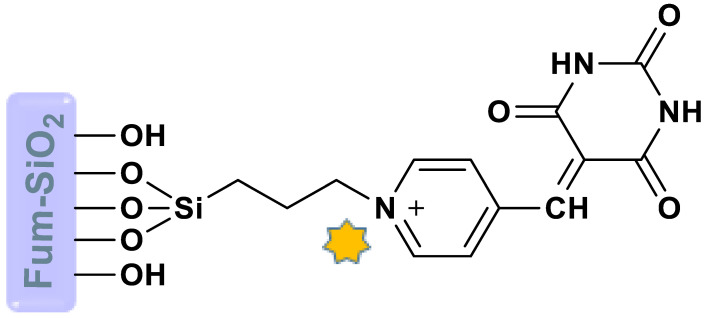	5.4 × 10^−3^ M for Hg^2+^ and 3.3 × 10^−3^ M for Cr_2_O_7_^2−^ ions	H_2_O:EtOH	-

## Data Availability

All data are available in Supporting Information file.

## Supplementary Materials

The following supporting information can be downloaded at: https://www.mdpi.com/article/10.3390/molecules29204825/s1, Figure S1. EDX of fumed-Si-Pr-Ald-Barb. Figure S2. Competition test of fumed-Si-Pr-Ald-Barb (2.5 mL, 0.02 g in 100 mL (H_2_O:EtOH/2:3)) for Hg²⁺ (200 µL, 1 × 10^−2^M) in the presence of other cations (λₑ_m_= 300 nm, λₑ_x_= 375 nm). Figure S3. The plot of flourescence intensity of fumed-Si-Pr-Ald-Barb against different concentrations of Hg²⁺. Figure S4. Competition test of fumed-Si-Pr-Ald-Barb (2.5 mL, 0.02 g in 100 mL (H_2_O:EtOH/2:3)) for Cr₂O₇^2−^ (200 µL, 1 × 10^−2^ M) in the presence of other anions (λₑ_m_= 300 nm, λₑ_x_= 380 nm). Figure S5. The plot of flourescence intensity of fumed-Si-Pr-Ald-Barb against different concentrations of Cr₂O₇²^−^. Scheme S1. Proposed binding mode between fumed-Si-Pr-Ald-Barb and Hg^2+^. Scheme S2. Proposed binding mode between fumed-Si-Pr-Ald-Barb and Cr_2_O_7_^2−^. Table S1. Mulliken charge distribution of selected atoms in Pr-Ald-Barb. Table S2. Estimated Mulliken atomic charges, Fukui functions and dual descriptor of selected atoms for Pr-Ald-Barb. Table S3. Quantum reactivity parameters of Pr-Ald-Barb, Pr-Ald-Barb+Hg^2+^, and Pr-Ald-Barb+Cr_2_O_7_^2−^ obtained from DFT.
