# Supplementary material for: Fumed-Si-Pr-Ald-Barb as a Fluorescent Chemosensor for the Hg2+ Detection and Cr2O72− Ions: A Combined Experimental and Computational Perspective
Source: Molecules. 2024 Oct 11;29(20):4825. doi: 10.3390/molecules29204825 (PMC11510132; doi:10.3390/molecules29204825)
Supplement: Supplementary file 1 [file molecules-29-04825-s001.zip › molecules-3211579-supplementary.pdf]

molecules

Supplementary Materials

Fumed-Si-Pr-Ald-Barb as a Fluorescent Chemosensor for the Hg^{2+} Detection and $Cr_2O_7^{2-}$ Ions: A Combined Experimental and Computational Perspective

Ghodsi Mohammadi Ziarani [1,*], Mahtab Rezakhani[1], Mehran Feizi-Dehnayebi [1,*], and Stoyanka Nikolova[2,*]

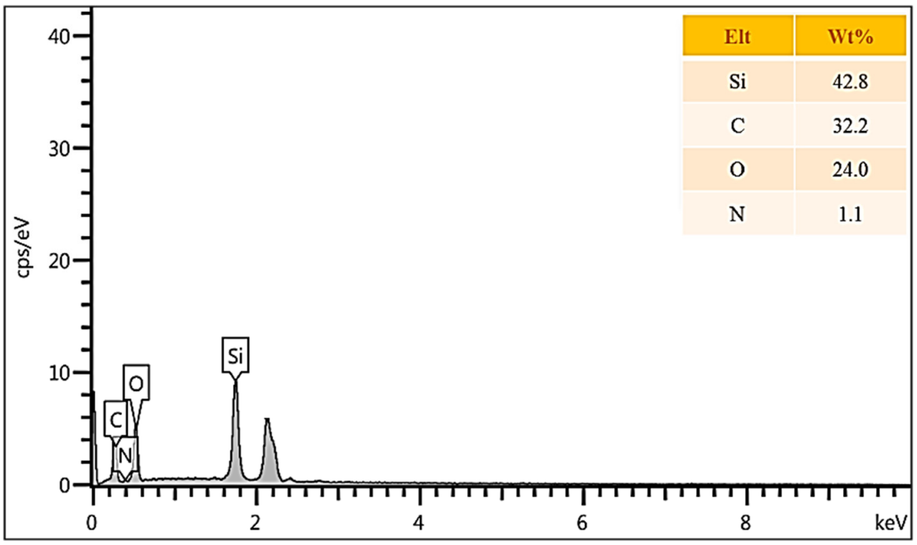

Elt	Wt%
Si	42.8
C	32.2
O	24.0
N	1.1

Figure S1. EDX of fumed-Si-Pr-Ald-Barb.

molecules

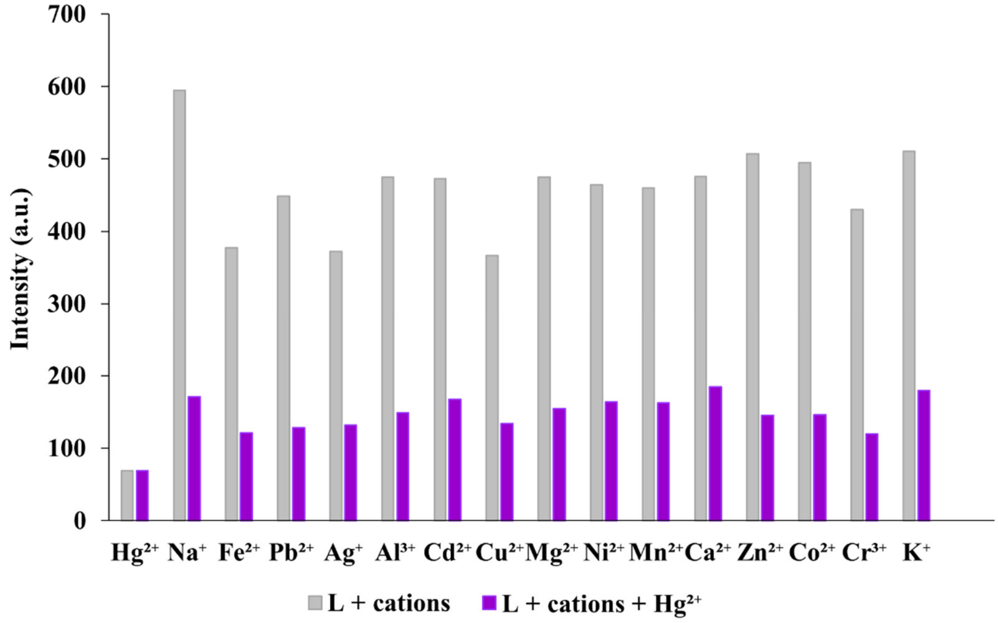

Figure S2. Competition test of fumed-Si-Pr-Ald-Barb (2.5 mL, 0.02 g in 100 mL (H_2O:EtOH/2:3)) for Hg^{2+} (200 μL, 1× 10^{-2}M) in the presence of other cations (λ_{em}= 300nm, λ_{ex}= 375nm).

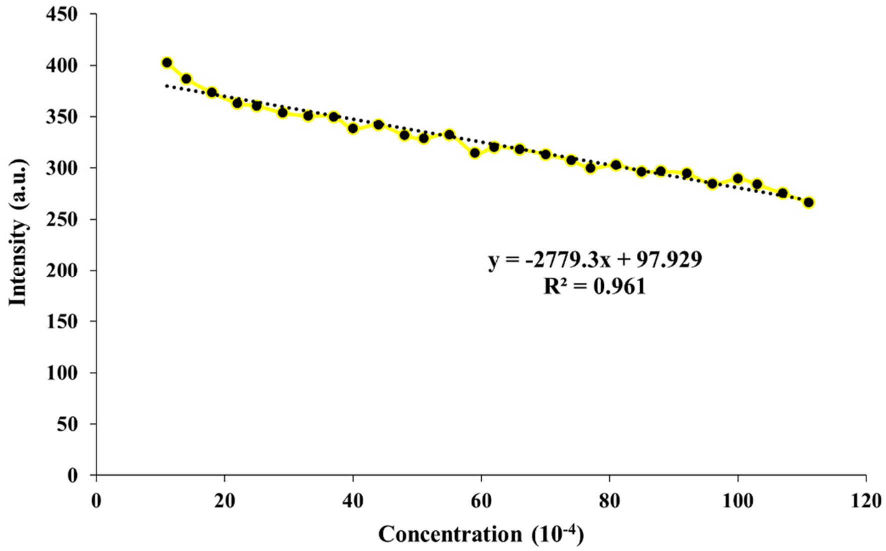

Figure S3. The plot of flourescence intensity of fumed-Si-Pr-Ald-Barb against different concentrations of Hg^{2+}.

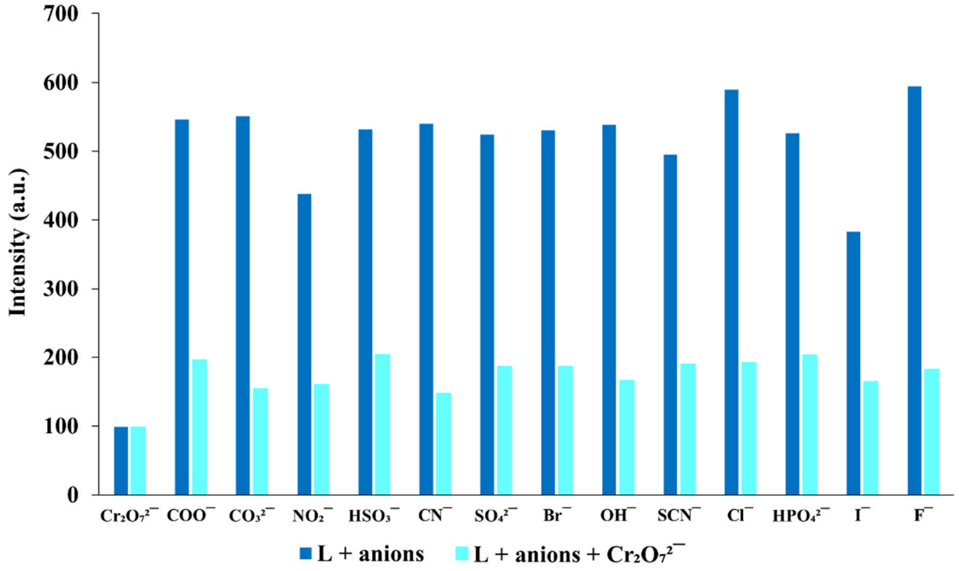

Figure S4. Competition test of fumed-Si-Pr-Ald-Barb (2.5 mL, 0.02 g in 100 mL (H_2O:EtOH/2:3)) for $Cr_2O_7^{2-}$ (200 μL, 1 × 10^{-2} M) in the presence of other anions (λ_{em}= 300 nm, λ_{ex}= 380 nm).

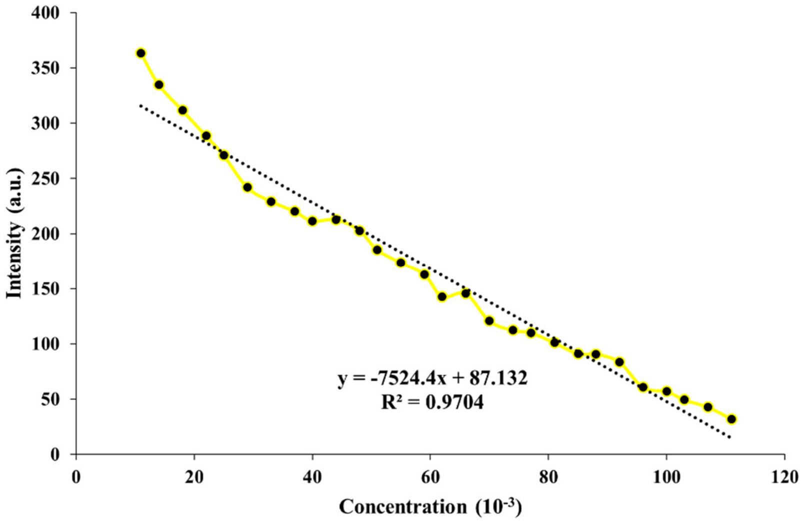

Figure S5. The plot of flourescence intensity of fumed-Si-Pr-Ald-Barb against different concentrations of $Cr_2O_7^{2-}$.

molecules

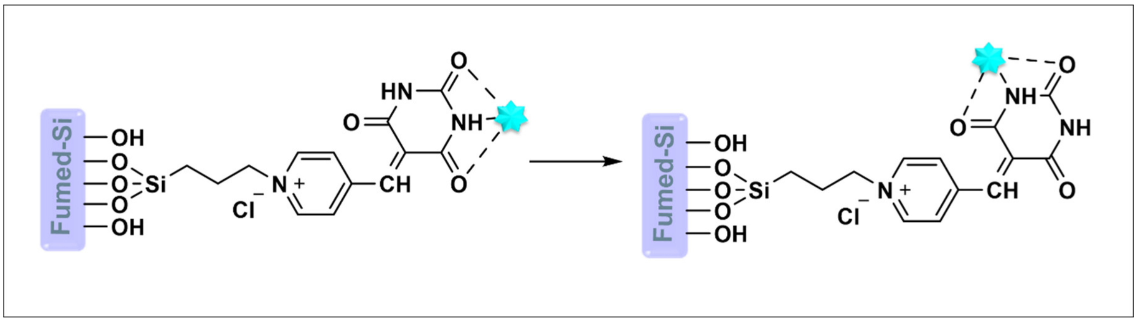

Scheme S1. Proposed binding mode between fumed-Si-Pr-Ald-Barb and Hg^{2+}.

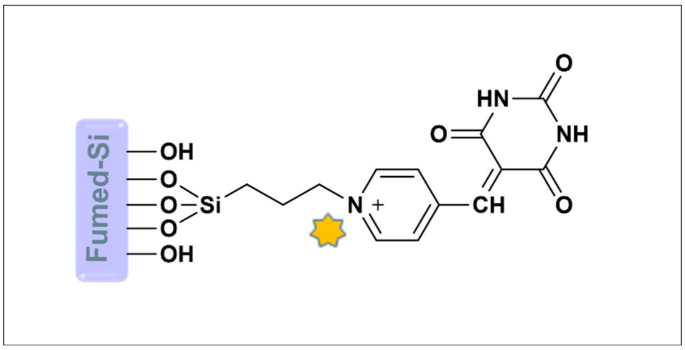

Scheme S2. Proposed binding mode between fumed-Si-Pr-Ald-Barb and $Cr_2O_7^{2-}$.

Table S1. Mulliken charge distribution of selected atoms in Pr-Ald-Barb.

Atom	Charge
**O1**	-0.481
**O2**	-0.472
**O3**	-0.463
**N1**	-0.336
**N2**	-0.340
**N3**	+0.414
**H_{-N1}**	+0.280
**H_{-N2}**	+0.282

Table S2. Estimated Mulliken atomic charges, Fukui functions and dual descriptor of selected atoms for Pr-Ald-Barb.

Atom	$q_{(N)}$	$q_{(N+1)}$	$q_{(N-1)}$	$\lvert f^+ \rvert$	$\lvert f^- \rvert$	Δf
**O1**	-0.481	-0.498	-0.446	0.017	0.035	-0.018
**O2**	-0.472	-0.487	-0.441	0.015	0.031	-0.016
**O3**	-0.463	-0.478	-0.433	0.015	0.030	-0.015
**N1**	-0.336	-0.349	-0.313	0.013	0.023	-0.010
**N2**	-0.340	-0.354	-0.314	0.014	0.026	-0.012
**N3**	+0.414	+0.444	+0.403	0.030	0.011	0.019
**H_{-N1}**	+0.280	+0.294	+0.273	0.014	0.007	0.007
**H_{-N2}**	+0.282	+0.295	+0.275	0.013	0.007	0.006

molecules

Table S3. Quantum reactivity parameters of Pr-Ald-Barb, Pr-Ald-Barb+Hg^{2+}, and Pr-Ald-Barb+$Cr_2O_7^{2-}$ obtained from DFT.

parameter	Pr-Ald-Barb	Pr-Ald-Barb+$Cr_2O_7^{2-}$	Pr-Ald-Barb+Hg^{2+}
E_{HOMO}	-5.31	-3.82	-5.31
E_{LUMO}	-4.61	-3.53	-4.68
$\Delta E_{(LUMO-HOMO)}$	0.70	0.29	0.63
χ	4.96	3.67	4.99
η	0.35	0.14	0.31
σ	2.85	7.14	3.22
P_i	-4.96	-3.67	-4.99
ω	35.14	48.10	40.16
